# Analysis of Pro- and Anti-Inflammatory Gene Response Patterns in Patients Receiving Phage Therapy

**DOI:** 10.3390/ijms27010172

**Published:** 2025-12-23

**Authors:** Hubert Kasprzak, Maciej Przybylski, Wojciech Fortuna, Sławomir Letkiewicz, Paweł Rogóż, Barbara Bubak, Andrzej Górski, Ryszard Międzybrodzki

**Affiliations:** 1 Bacteriophage Laboratory, Phage Therapy Department, Hirszfeld Institute of Immunology and Experimental Therapy, Polish Academy of Sciences, 53-114 Wrocław, Poland; 2 Chair and Department of Medical Microbiology, Medical University of Warsaw, 02-004 Warsaw, Poland; 3 Phage Therapy Unit, Hirszfeld Institute of Immunology and Experimental Therapy, Polish Academy of Sciences, 53-114 Wrocław, Poland; wojciech.fortuna@hirszfeld.pl (W.F.);; 4 Medical Faculty, Katowice Business University, 40-659 Katowice, Poland; 5 Department of Clinical Immunology, Medical University of Warsaw, 02-006 Warsaw, Poland

**Keywords:** bacteriophages, phage therapy, immune response, gene expression, pro-inflammatory genes, anti-inflammatory genes

## Abstract

Phage therapy (PT) is a promising alternative for antibiotic-resistant infections, but its immunomodulatory effects in clinical settings remain poorly understood. This exploratory observational study aimed to characterize pro- and anti-inflammatory gene response patterns in ten patients undergoing personalized PT at the Phage Therapy Unit in Wrocław. Peripheral blood mononuclear cells (PBMCs) and granulocytes were analyzed to assess changes in the expression of 22 selected immune-related genes associated with innate and adaptive immune signaling pathways. While no uniform pattern of immune gene expression was observed across the cohort, individual cases exhibited significant up- or downregulation of specific genes. Interestingly, we identified biological age as a potential determinant of the host response. Specifically, older patients showed higher activation of the innate sensing machinery in PBMCs, characterized by a higher *TLR4* fold change which may reflect the “inflammaging” phenomenon. These findings suggest that chronic exposure to bacterial viruses (bacteriophages), unlike many viral infections, does not trigger a predictable, significant systemic immune activation and that immune responses to PT are highly individualized by host- and phage-related biological factors. By documenting this spectrum of real-world responses, our work provides baseline data and hypotheses to guide the rational design of future preclinical and clinical investigations.

## 1. Introduction

In recent years, the impact of viral infections on the expression of immune response-related genes has been intensively studied [1,2,3,4,5,6,7]. The results of these studies present a consistent picture, at least concerning viruses that cause acute infections. Infections with COVID-19, RSV, HCV, HHV-6, adenoviruses, HSV-1, and influenza A virus, strongly stimulate pro-inflammatory response, as confirmed by both in vitro and in vivo studies. In contrast to the well-documented inflammatory responses elicited by pathogenic human viruses, the immunological effects of bacteriophages are less clear. As viruses that exclusively infect bacteria, their direct impact on host cells is not pathogenic, yet their presence within the human body raises important questions about their potential to modulate the immune system [8].

Phage therapy (PT) is an alternative approach to combating bacterial infections, particularly in cases where antibiotic resistance limits conventional treatment options. While bacteriophages have been studied primarily for their bactericidal properties, their potential effects on the human immune system remain largely unexplored [9,10,11]. Moreover, accumulating evidence indicates that phages can interact with eukaryotic cells and modulate immune cell activity [12,13,14].

Although bacteriophages were long considered exclusively bacterial pathogens, accumulating evidence demonstrates that they can interact with mammalian cells through multiple mechanisms. These interactions may occur at the cell surface—via recognition of bacteriophage components by pattern recognition receptors (PRRs) or through molecular mimicry of host receptors such as polysialic acid [15], as well as through endocytic processes including non-specific macropinocytosis [12]. Importantly, immune activation can result both from extracellular sensing of phage-associated molecular patterns and from intracellular recognition following endocytosis. Mammalian cells can internalize bacteriophages via endocytic pathways [16], which can lead to the induction of pro- and anti-inflammatory cytokines [17]. In parallel, exposure of the immune system to phage antigens, regardless of whether internalization occurs, can drive the development of anti-phage antibodies through adaptive immune activation [18]. For example, purified DNA from *Caudovirales* phages has been shown to elicit a robust interferon-gamma (IFN-γ) response in immune cells [19], and fluorescence microscopy in the same study demonstrated dendritic-cell uptake of phage particles, facilitating antigen presentation and immune recognition. During PT, bacteriophages may therefore influence not only their bacterial targets but also host immune processes [9]. The magnitude and direction of these effects are complex and highly context-dependent, shaped by factors such as phage preparation purity, route of administration, and pre-existing anti-phage immunity [8,20,21].

Given the growing interest in phage therapy and the still-limited knowledge of its interactions with the human immune system in vivo, there is an urgent need to characterize these effects in a clinical setting. Therefore, the aim of this observational study is to describe and characterize the profiles of pro- and anti-inflammatory gene responses in patients undergoing PT. By assessing the expression of selected immune-related genes in peripheral blood mononuclear cells (PBMCs) and granulocytes, we seek to determine whether chronic exposition to bacterial viruses induces any significant immunological shifts and to explore their potential associations between the observed clinical outcomes.

This work, conducted as an exploratory investigation within an established experimental treatment program for patients who provided consent, is intended to generate hypotheses for future controlled studies. Understanding these responses is crucial for evaluating both the safety and potential immunomodulatory effects of PT.

## 2. Results

### 2.1. Patient Characteristics

A total of ten patients (5 females and 5 males), with a median age of 47.5 years (range: 21–79), were enrolled in the study. All presented with chronic, antibiotic-refractory bacterial infections, predominantly involving *Staphylococcus aureus*, *Pseudomonas aeruginosa* and *Escherichia coli* (Table 1). Phage therapy was administered for 13–29 days *via* oral, topical, and/or inhalation routes, according to the site of infection.

The clinical response to PT varied across the cohort. Based on the clinical assessment scale, beneficial therapeutic effects (C score) were observed in 40% of patients, consistent with our previous findings in a larger patient cohort [22]. Conversely, one patient demonstrated equivocal improvement, one showed only transient benefit, three did not respond to treatment, and one experienced clinical deterioration.

Regarding systemic inflammatory markers, changes in C-reactive protein (CRP) and erythrocyte sedimentation rate (ESR) measured before and during PT were strongly positively correlated (*rs* = 0.89; *p* = 0.007), and both showed significant negative correlations with the clinical outcome of PT (CRP: *rs* = −0.75, *p* = 0.033; ESR: *rs* = −0.80, *p* = 0.017).

### 2.2. Gene Expression Fold Change and Correlations

In order to comprehensively assess the immunomodulatory effects of phage therapy, a gene panel was selected to analyze key aspects of the immune response. The chosen genes can be divided into several main functional groups:

1. Innate immunity receptors and signaling molecules—the panel includes genes encoding pattern recognition receptors, such as Toll-like receptor 2 (TLR2) and Toll-like receptor 4 (TLR4), as well as the intracellular sensor nucleotide-binding oligomerization domain containing 1 (NOD1), which are crucial for initiating the response to bacterial components. The expression of genes for central signal transduction molecules, myeloid differentiation primary response 88 (MYD88) and nuclear factor kappa-light-chain-enhancer of activated B cells (NFκB), as well as the modulatory Toll-like receptor 10 (TLR10), was also analyzed.
2. Cytokines and chemokines shaping the inflammatory response—to evaluate the nature and balance of the immune response, the panel included genes for cytokines with diverse functions:
  - Th2-response cytokines, such as interleukin-4 (IL-4), interleukin-5 (IL-5), and interleukin-13 (IL-13), which are central to type 2 immunity. IL-4 and IL-13 are essential in the repair of injured tissues, for instance, by activating macrophages and epithelial cells; however, excessive or uncontrolled activity leads to pathological fibrosis. IL-5 is the main factor responsible for recruitment, activation, and promotion of eosinophil survival at the sites of inflammation; therefore, assessing its expression allows for the analysis of the eosinophilic component that is crucial to the pathogenesis of many allergic and inflammatory diseases.
  - Pro-inflammatory and cell-mediated response cytokines—the panel included genes for key pro-inflammatory cytokines such as tumor necrosis factor (TNF), interleukin-1 beta (IL-1β), interleukin-6 (IL-6); cytokines associated with T-cell responses such as interferon-gamma (IFN-γ), interleukin-17A (IL-17A), interleukin-2 (IL-2), and interleukin-21 (IL-21); an important neutrophil chemoattractant, C-X-C motif chemokine ligand 8 (CXCL8); as well as the regulatory interleukin-10 (IL-10) and growth differentiation factor 15 (GDF15).
3. Effector molecules and growth factors—the panel was supplemented with genes encoding important effector molecules, including the antimicrobial protein defensin beta 1 (DEFB1) and the enzyme myeloperoxidase (MPO), as well as growth and differentiation factors for immune cells, such as colony-stimulating factor 2 (granulocyte-macrophage) (CSF2).

This multi-faceted approach allows for a holistic analysis of the immunological shifts occurring in PBMCs and granulocytes of patients undergoing phage therapy.

The mean and median FC values for gene expression are presented in Table 2. For several genes, the mean FC was ≥2, primarily in PBMCs (*IL1B*, *IL17A*, *IL2*, *IL6*, *IL21*, *CXCL8*, *MPO*, *GDF15*, *DEFB1*, *IL5*, *IL13*, *IL4*, *IL10*) compared to granulocytes (*IFNG*, *MPO*, *GDF15*, *IL4*). However, the median values for nearly all genes fell between 0.5 and 2 (except for *IL2* and *CXCL8* in PBMCs), and they did not follow a normal distribution. In consequence, no statistically significant differences in gene expression were found between samples collected before and during PT for either PBMCs or granulocytes when a non-parametric test was used. Similarly, no statistically significant differences were detected based on gender (male vs. female), pathogen type (Gram-negative bacteria such as *Pseudomonas aeruginosa*, *Escherichia coli*, *Enterobacter hormaechei*, and *Klebsiella pneumoniae vs. Gram-positive* bacteria such as *Staphylococcus aureus* vs. mixed Gram-negative/Gram-positive infections), or route of phage administration (oral/intrarectal vs. topical/inhalation/nasal drops vs. combined), but these subgroup analyses were likely underpowered due to the limited number of patients in each category, warranting a cautious interpretation of these findings.

No significant correlations were observed between FC values of any analyzed immune-related genes and clinical outcomes of PT. A significant moderate/high positive correlation (Spearman’s *rs* between 0.67 and 0.77) was observed between the increase in WBC count during PT and changes in the expression of *GDF15*, *NOD1*, *CSF2*, *MPO*, *IL10*, *IL6*, and *IL21* in granulocytes. Other significant correlations of gene expression fold changes with clinical parameters included:

- PT duration positively correlated with *NOD1* in PBMCs (*rs* = 0.67, *p* = 0.034), CSF2 in PBMCs (*rs* = 0.71, *p* = 0.02), and *MYD88* in granulocytes (*rs* = 0.67, *p* = 0.034);
- PT duration negatively correlated with *GDF15* in granulocytes (*rs* = −0.75, *p* = 0.012), *NFKB1* in granulocytes (*rs* = −0.65, *p* = 0.041), *CSF2* in granulocytes (*rs* = −0.73, *p* = 0.016), and *MPO* in granulocytes (*rs* = −0.81, *p* = 0.004);
- patients’ age correlated positively with *NOD1* in PBMCs (*rs* = 0.65, *p* = 0.043), and *CSF2* in PBMCs (*rs* = 0.70, *p* = 0.025);
- patients’ age correlated negatively with *NFKB1* in granulocytes (*rs* = −0.83, *p* = 0.029).

Recognizing that biological age is a critical determinant of immune plasticity and baseline inflammation—a phenomenon known as “inflammaging” [23,24]—we stratified the cohort into two age-dependent groups: a younger group (age 21–38, median 27 years, *n* = 5) and an older group (age 57–79, median 67 years, *n* = 5). Indeed, this revealed a statistically significant upregulation of *TLR4* in the older patients (median FC values were 0.89 and 1.37, respectively; *p* = 0.016) within PBMCs. Furthermore, *MYD88*, the key downstream adaptor for TLR signaling, showed a trend towards upregulation (median FC was 0.78 and 1.33 in the younger and in older groups, respectively; *p* = 0.056). Together, these findings may indicate a coordinated elevation of the innate sensing machinery in older patients. Although these FCs appear modest compared to the high-magnitude induction typical of downstream cytokines, they act as a critical ‘sensitizing’ mechanism [25]. As upstream regulators, *TLR4* and *MYD88* operate near the top of the signaling hierarchy, where even slight changes in receptor density can substantially lower the activation threshold. Through intracellular signal amplification, this constitutive upregulation primes the immune system for hyper-reactivity, which is the hallmark of the chronic, low-grade inflammation observed in the inflammaging phenotype.

The same analytical approach was applied to compare transcriptional shifts between the short-term therapy group (days 13–14 of PT; median day 14; *n* = 4) and the long-term group (days 21–29 of PT; median day 21.5; *n* = 6). However, no significant differences in FC values for any gene in either PBMCs or granulocytes were shown.

The transcriptional patterns observed in this cohort appear to reflect a complex adaptive remodeling of innate immune function in the context of long-standing bacterial infection rather than a direct or uniform immunomodulatory effect of phage therapy. Given that the observation period during PT ranged from only 13 to 39 days, whereas the underlying infections had been present for several months, the absence of significant correlations between PT duration and gene expression changes argues strongly against a time-dependent immunological impact of phages. Instead, the coexistence of increased expression of upstream innate-sensing mediators—such as *NOD1* in PBMCs and *MYD88* in granulocytes—with a parallel downregulation of downstream effector and inflammatory transcripts in granulocytes (including *MPO*, *NFKB1*, *CSF2*, and *GDF15*) is more consistent with mechanisms described in chronic infection-associated adaptation of innate immunity [26,27].

This pattern—characterized by sustained or heightened signaling sensitivity coupled with attenuated effector output—aligns with concepts of immune tolerance and regulated host–pathogen coexistence, in which prolonged exposure to microbial PAMPs drives a recalibration of the innate response to prevent excessive inflammation while maintaining pathogen surveillance [26,28]. Although these findings do not exclude the possibility that phage therapy contributes modestly to the overall immune milieu, the available data do not support a distinct or dominant phage-specific transcriptional signature. Rather, the gene expression landscape captured in this study most plausibly represents the immune system’s ongoing accommodation to chronic bacterial infection, with PT superimposed on—and not fundamentally reshaping—this pre-existing adaptive state [27].

**Figure 1:**
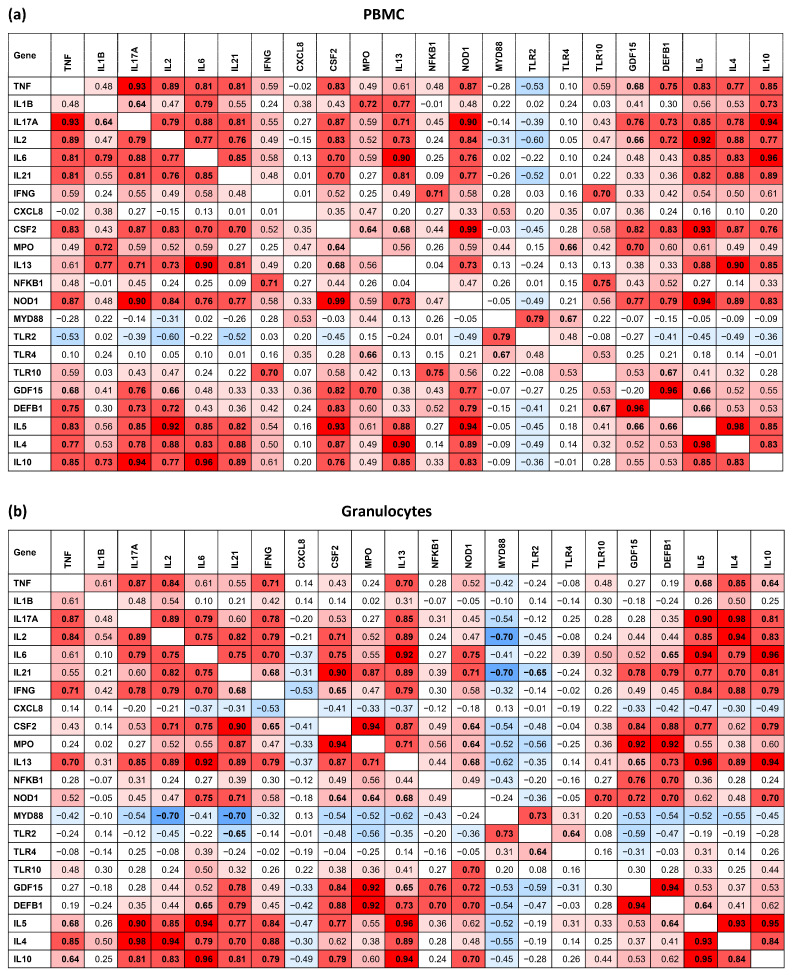
Correlation of FC values in gene expression in PBMCs (**a**) and granulocytes (**b**) isolated from 10 patients undergoing experimental phage therapy. Significant Spearman’s correlation coefficient values (*p* < 0.05) are highlighted in bold. Different background colors of the cells correspond to the strength of the correlation, ranging from blue for highly negative, through gray for insignificant, to red for very highly positive.

We also identified several potential relationships between changes in the expression of all studied genes (Figure 1). The observed co-expression profiles in both cell populations are indicative of robust, interconnected gene networks. These networks functionally link the expression of pro-inflammatory and regulatory cytokines with innate immune recognition receptors, key signaling molecules, and antimicrobial effectors, reflecting a physiologically coherent immunological response. For example, in PBMCs (Figure 1a), a major network of strong mutual correlations was observed among a broad range of cytokine and immune-related genes, including *TNF*, *IL17A*, *IL2*, *IL6*, *IL21*, *IL5*, *IL4*, *IL10*, *CSF2*, and the innate immune receptor *NOD1*. The expression of *IL1B* was also positively correlated with a key subset of these genes, namely *IL17A*, *IL6*, *IL10*, and *IL13*. This indicates a coordinated, systemic upregulation of key mediators driving both inflammatory and regulatory responses. Furthermore, we observed strong correlations reflecting the activation of specific innate immune signaling cascades. The expression of the adaptor protein *MYD88* was positively correlated with that of *TLR2* and *TLR4*. In addition, we identified a distinct axis of co-expression involving *IFNG*, *NFKB1*, and *TLR10*, which appears to reflect the integration of established signaling dependencies. The interplay between IFN-γ signaling and NF-κB activation is a cornerstone of pro-inflammatory responses [29]. Furthermore, the expression of *TLR10* is known to be mechanistically dependent on the NF-κB pathway in response to inflammatory stimuli [30]. Our observation is therefore consistent with an intrinsic regulatory circuit where a primary inflammatory signal (*IFNG*) acts through a key mediator (*NFKB1*) to simultaneously upregulate *TLR10*, a receptor implicated in the subsequent modulation of the immune response [31,32].

The expression of genes encoding effector molecules was tightly integrated with cytokine and signaling pathways. Myeloperoxidase (*MPO*) expression correlated positively with *IL1B*, *CSF2*, *TLR4*, and *GDF15*. Furthermore, the growth differentiation factor *GDF15* and the antimicrobial peptide *DEFB1* were mutually correlated with a large group of pro-inflammatory genes (*TNF*, *IL17A*, *IL2*, *IL5*, *CSF2*, and *NOD1*), while *GDF15* also correlated with *MPO* and *DEFB1* with *TLR10*.

In granulocytes, the analysis revealed similarly robust, albeit distinct, patterns of gene co-expression (Figure 1b). A vast network of mutually positive correlations was identified among key cytokine genes, including *TNF*, *IL17A*, *IL2*, *IL6*, *IL21*, *IFNG*, *IL5*, *IL13*, *IL4*, *IL10*, and *CSF2* (with specific exceptions as noted). This highlights a broad and coordinated cytokine response within this cell population. A significant hub of mutual correlations was centered on genes critical for effector functions and signaling, specifically *IL13*, *IL21*, *CSF2*, *MPO*, *NOD1*, *GDF15*, and *DEFB1*. This suggests a tightly regulated module linking cytokine signaling directly to degranulation, innate sensing, and antimicrobial activity. This co-activation of pathways was further supported by specific correlations: *NFKB1* expression was linked to *GDF15* and *DEFB1*, while the innate sensor *NOD1* correlated with the cytokine genes *IL6* and *IL10*, as well as with *TLR10*. Finally, canonical TLR signaling was evident in the positive correlations between *MYD88* and *TLR2*, and between *TLR2* and *TLR4*.

### 2.3. Hierarchical Gene Clustering

**Figure 2:**
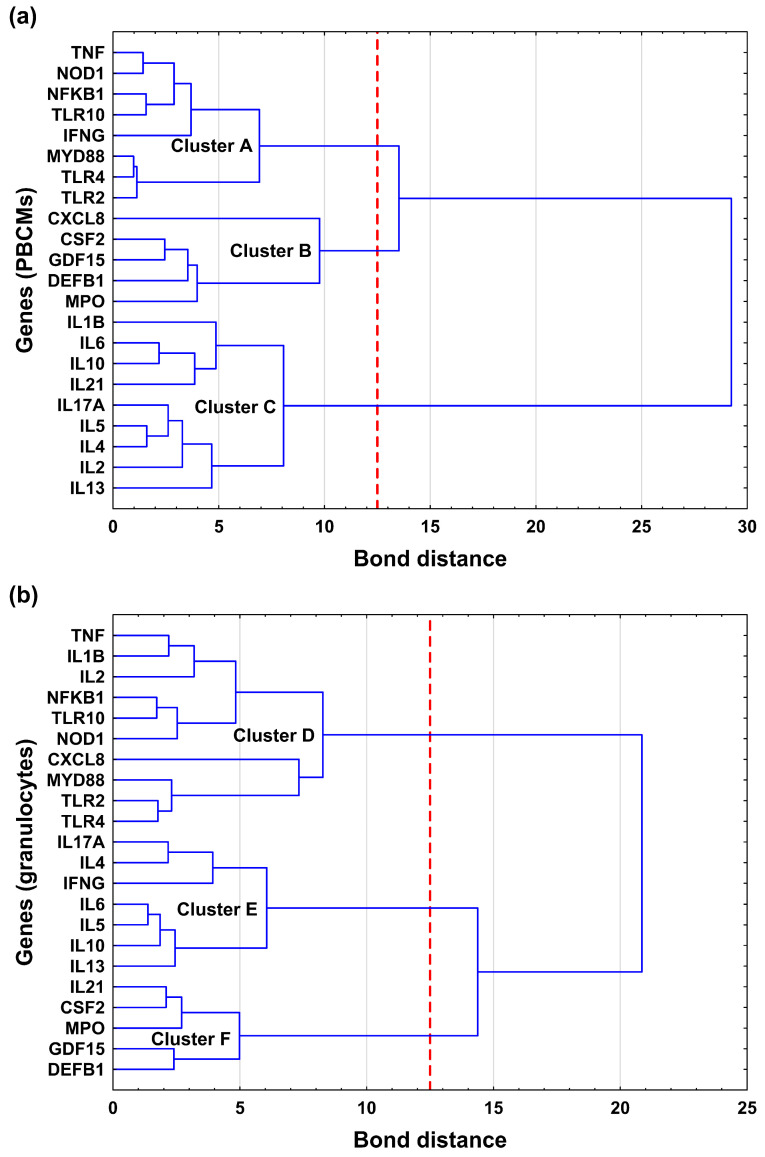
Hierarchical clustering of gene expression profiles in PBMCs (**a**) and granulocytes (**b**). The dendrogram was constructed using Ward’s minimum variance method with Euclidean distance, based on log2FC values for the *analysed* genes. The red dashed line indicates the cut-off threshold used to define clusters.

To identify robust transcriptional networks tailored to distinct cellular compartments, hierarchical clustering was performed independently for PBMCs and granulocytes (Figure 2). This approach was adopted to capture cell-type-specific co-expression patterns that might otherwise be masked in an aggregated dataset due to the fundamental physiological differences between mononuclear and polymorphonuclear lineages. This analysis revealed compartment-specific regulatory architectures.

In PBMCs (Figure 2a), we observed a strict functional segregation. The adaptive and regulatory cytokine genes (*IL1B*, *IL6*, *IL10*, *IL17A*, *IL2*, *IL4*, *IL5*, *IL13*, *IL21*) formed a distinct, isolated module (cluster C), clearly separated from the innate sensing machinery (*TLR2*, *TLR4*, *TLR10*, *NOD1*) and primary signaling mediators (*NFKB1*, *MYD88*, *TNF*, *IFNG*) which represent the cellular “command center” for pathogen detection (cluster A). Third cluster (B) comprised a set of genes related to innate immune *defence* and leukocyte recruitment (*MPO*, *DEFB1*, *GDF15*, *CXCL8*, *CSF2*), whose expression within PBMCs is predominantly attributable to the monocyte subpopulation.

In contrast, granulocytes displayed a more integrated “rapid-response” topology (Figure 2b). Potent pro-inflammatory cytokine genes (*TNF*, *IL1B*, *IL2*) clustered tightly with innate sensor genes (*TLR2*, *TLR4*, *NOD1*), the central adaptor *MYD88*, and the downstream transcription factor *NFKB1* (Cluster D). Notably, the neutrophil chemoattractant *CXCL8* also shifted into this module, indicating a tightly coupled network in which pathogen sensing is directly linked to immediate inflammatory signaling and auto-recruitment. This arrangement suggests that, in granulocytes, the transcriptional machinery responsible for detection and early response operates as a single functional unit, enabling rapid and coordinated activation. Cluster E encompassed cytokine genes associated with modulation of adaptive immune responses (*IL17A*, *IL4*, *IL5*, *IL6*, *IL10*, *IL13*, *IFNG*). Finally, Cluster F formed a discrete group enriched for genes essential for granulocyte effector function and survival (*MPO*, *DEFB1*, *GDF15*, *CSF2*, *IL21*).

As suggested, this clustering approach aligns well with the observed FC correlations across various genes. However, we believe it better reflects the underlying functional dependencies. For instance, in PBMCs, *TNF* and *NOD1* are assigned to Cluster A, even though their FC values strongly correlate with those of interleukins grouped in Cluster C. Interestingly, in contrast to interleukins, which are primarily secreted products of leukocytes, most genes in Cluster A represent intracellular sensors or signaling molecules that can also be expressed by lymphoid cells.

### 2.4. Inter-Patient Variation in FC Expression Profiles

**Figure 3:**
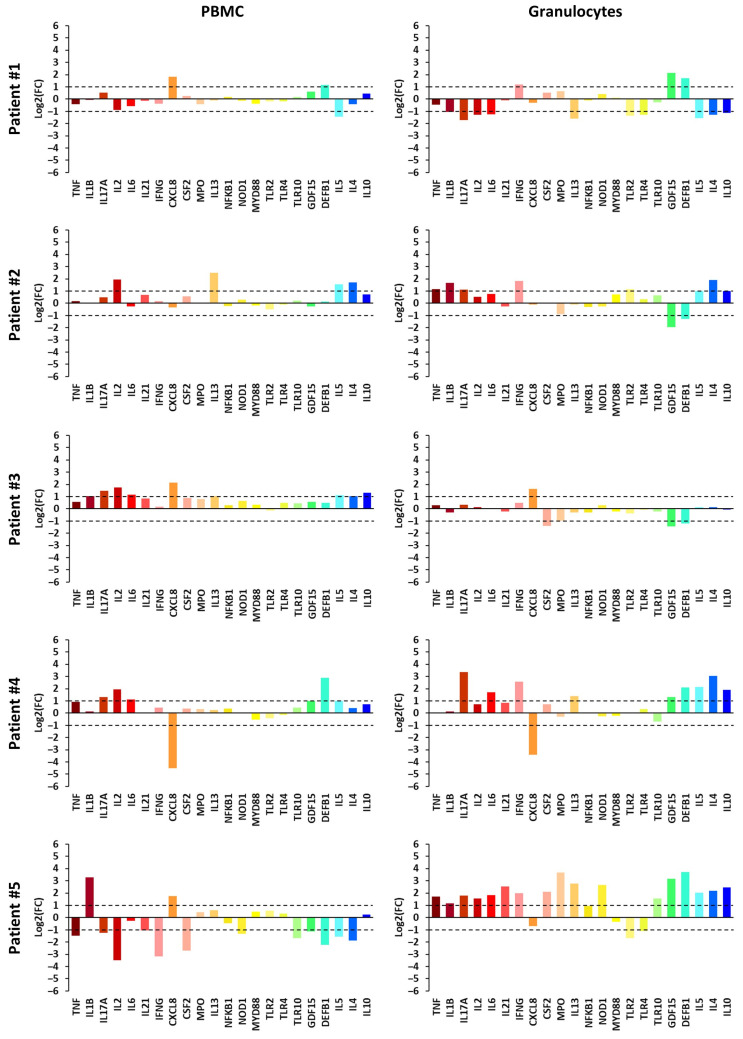
Changes in gene expression (log_2_FC) in patients No. 1–5 during phage therapy.

**Figure 4:**
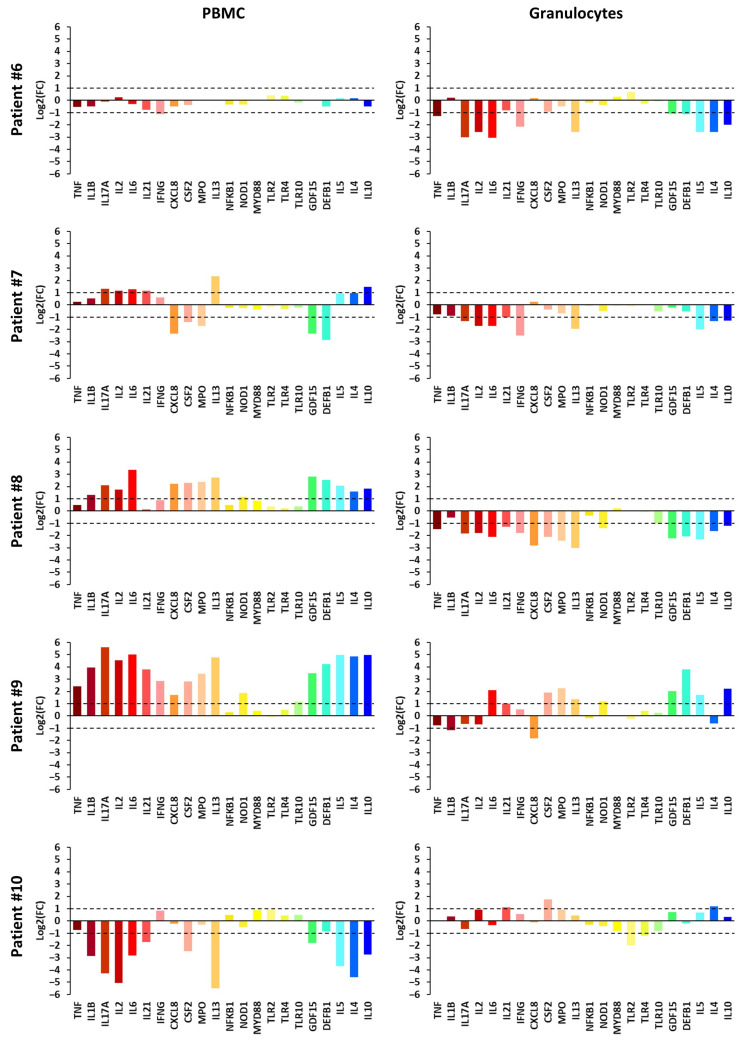
Changes in gene expression (log_2_FC) in patients No. 6–10 during phage therapy.

The individual transcriptional landscapes shown in Figure 3 and Figure 4, which display log_2_FC values to facilitate interpretation of differences in specific gene expression, illustrate how the co-regulatory patterns identified by stratified analysis, correlation matrices, and hierarchical clustering manifest at the patient level. In PBMCs, for example, *TNF*, *IL6*, *IL10* displayed tightly synchronized behavior: in patients with pronounced immune activation (#9) or suppression (#10), these genes shifted in parallel, whereas in quiescent profiles (#1, #2, #3) the entire module remained near baseline. A similar relationship emerged in granulocytes, where correlations linking the innate sensor *NOD1* with effector molecules such as *MPO* and *GDF15* were reflected in patient #4, whose “rapid-response” pattern showed concurrent elevation of sensing and antimicrobial effector genes. Furthermore, the baseline activity of these modules appears to be modulated by clinical covariates identified in our correlation analysis; specifically, the age-dependent “priming” of innate sensors (*TLR4*, *NOD1*) and the therapy-duration-dependent modulation of signaling adapters (*MYD88*) suggest that the sensitivity of these gene clusters is dynamically tuned by host biological factors. Taken together, these patient-level examples indicate that the statistical associations among pro-inflammatory mediators were not artifacts of cohort-level aggregation but instead arose from coordinated gene-module behavior within individuals.

Negligible FC values for almost all analyzed genes in both PBMCs and granulocytes were observed in three patients (#1, #2, and #3). Granulocytes from patients #4 and #5 showed quite similar FC profiles, with a slight increase in gene expression (FC between 2 and 10) for 9 and 17 genes, respectively, while no comparable pattern was observed in their PBMCs. A small decrease in gene expression (FC between 0.1 and 0.5) for over half of the studied genes was observed in granulocytes from patients #6, #7, and #8. However, their PBMCs showed distinct patterns: patient #6 displayed no significant FC values; patient #7 showed a mixed pattern of slight increases or decreases in expression of some genes; and patient #8 exhibited a slight overall increase in the expression of most genes. The most pronounced changes were observed in the PBMCs of patients #9 and #10. PBMCs from patient #9 were highly activated (FC > 10 for 12 genes), with only slight activation of some genes in granulocytes. In contrast, patient #10 showed a marked decrease (FC < 0.01 for 5 genes) in the expression of several genes, indicating strong transcriptional repression during PT.

To capture the biological reality obscured by aggregate data, we analyzed specific patient cases that illustrate distinct immunophenotypes in association with clinical outcomes.

A pattern of transcriptional “silence” was observed in patients #1, #2, and #3, who exhibited negligible changes in gene expression despite ongoing infection. Notably, patient #3 achieved clinical improvement without evidence of systemic immune modulation. This profile may indicate a lack of systemic spillover, consistent with effective local phage-mediated bacterial lysis that remains undetectable in peripheral blood. This highlights that clinical efficacy does not necessarily require systemic immune activation—a phenomenon potentially further modulated by the specific immunogenicity of the phages employed [33].

A unique ‘active defense’ profile was observed in patient #4 (atopic dermatitis superinfected with *S. aureus*). Unlike the quiescence observed in other responders, this patient mounted a robust response characterized by the upregulation of antimicrobial effectors *DEFB1* and *GDF15*. This is consistent with the cytokine’s known role in boosting antimicrobial immunity [34]. Crucially, this upregulation was accompanied by a marked downregulation of *CXCL8* in both PBMCs and granulocytes. Given the established role of the chemokine CXCL8 in driving neutrophil recruitment and exacerbating inflammation in atopic dermatitis, this specific reduction provides a possible molecular basis for the observed clinical relief in this case [35].

In patient #5 with Kartagener’s syndrome and chronic bronchial *P. aeruginosa* infection, we observed a clinically significant increase in WBC count and CRP levels, despite no detectable change in physical symptoms of infection. Interestingly, control microbiological analysis of a sputum sample obtained on the day of blood collection during PT, as well as another sample collected during follow-up, revealed the presence of *S. aureus*. This unexpected scenario—eradication of the target pathogen with the emergence of a new one—may explain why the rise in inflammatory markers was accompanied by widespread activation of genes in granulocytes. In these patients, granulocytes constitute the first, albeit functionally suboptimal, line of antibacterial defense, and this was paralleled by a distinct increase in *IL1B* expression in PBMCs, suggesting stimulation of the immune response to this new bacterial pathogen.

Despite sharing the same pathogen (*S. aureus*), cases #6 and #8 displayed divergent transcriptional trajectories consistent with their distinct clinical outcomes. Patient #6 (chronic rhinosinusitis), who achieved clinical improvement, showed a profile of immune resolution characterized by concurrent downregulation of *GDF15* and *DEFB1*, signaling a return to homeostasis [34]. In contrast, patient #8 suffering from surgical site infection with diabetes presented a discordant profile, where systemic activation in PBMCs was paired with broad granulocyte suppression. This signaling incoherence, associated with questionable clinical improvement, reflects impaired tissue repair mechanisms often dependent on balanced type 2 cytokine signaling [36].

A discordant signature was observed in patient #7, who was infected with *P. aeruginosa* and exhibited suboptimal clinical outcomes (transient improvement only). This patient presented a “mixed” profile characterized by upregulation of *IL13* and downregulation of *CXCL8* in PBMCs, against a background of broad granulocyte suppression. In this context, the observed downregulation of *CSF2* could be consistent with *P. aeruginosa*-mediated modulation [37], while the concurrent downregulation of *GDF15* may correlate with a stalled recovery phase [34,38].

The full spectrum of responses in our cohort was characterized by two extremes. Patient #9, who experienced clinical deterioration manifested by worsening inflammation of the lower-leg ulcer, represented the hyperinflammatory extreme, exhibiting massive upregulation of *TNF*, *IL1B*, *IL6*, and *IL17A* in PBMCs. This transcriptional surge was disproportionate to the modest rise in standard systemic markers (CRP: 2.35 to 5.39 mg/dL). In contrast, patient #10 (clinical improvement) showed downregulation of both pro-inflammatory cytokines and the regulatory IL-10, illustrating a profile of immune resolution and homeostasis restoration rather than a specific ‘phage signature’.

## 3. Discussion

### 3.1. Immunologically ‘Quiet’ Systemic Transcriptional Response to Therapeutic Phages

Evidence dating back for several decades has consistently demonstrated that pathogenic viruses typically acts as a potent inducer of systemic pro-inflammatory responses. For instance, severe infections with respiratory syncytial virus (RSV) provoke a broad cytokine response involving both Th1 and Th2 pathways [39]. Similarly, transcriptomic analyses of patients infected with human herpesvirus 6 or adenovirus type 7 reveal extensive activation of innate immunity, characterized by the upregulation of TLR signaling (e.g., *TLR1*, *TLR6*, *TLR8*, *MYD88*) and elevated levels of pro-inflammatory mediators like TNF and IL-6 [40,41]. In severe cases, such as SARS-CoV-2, this activation can escalate to a damaging cytokine storm driven by TLR4-mediated signaling [42].

In contrast to these pathogenic dynamics, the data presented here do not indicate that our therapeutic bacteriophages trigger an analogous, universal “viral alarm” signal. It is critical that our cohort consists of patients with chronic, often recalcitrant bacterial infections, whose immune systems are already engaged and potentially dysregulated. In this context, the primary concern is whether the exposition to high-titer phage preparations acts as an additional inflammatory stressor. Our findings indicate otherwise, as we observed neither the activation of TLR signaling cascades typically driven by acute viral challenges nor the upregulation of *IFNG*. This absence of a uniform, high-magnitude inflammatory signature supports the interpretation that therapeutic phages do not impose an additive inflammatory burden on the host, even in patients with pre-existing immune activation due to chronic bacterial infection. Nevertheless, these findings align with recent clinical safety data. For instance, Chan et al. [43] reported that nebulized phage therapy applied up to 10 days was generally well tolerated in nine cystic fibrosis adults. While subjective fevers were noted in some patients, no adverse events related to severe systemic immunological reactions were observed. Similarly, Ooi et al. [44] reported that intranasal bacteriophage irrigations administered to nine patients with chronic rhinosinusitis were safe and well tolerated, with no serious adverse events attributed to the treatment [44].

Consistent with preclinical evaluations demonstrating the safety of lytic phage lysates in invertebrate models such as *Galleria mellonella*, our transcriptomic data in humans corroborate this benign profile [45]. Whereas the larval model showed an absence of intrinsic toxicity and endotoxin-mediated shock during 96 h exposure to phage lysates, our study extends these findings to the clinical setting, demonstrating an immunologically “quiet” transcriptional landscape even under substantially higher and more prolonged exposure to therapeutic phages. Moreover, mechanistic studies in animal models of *A. baumannii* pneumonia indicate that effective phage therapy significantly downregulates pro-inflammatory genes (*TNF*, *IL6*, *IL1B*) following bacterial clearance. Crucially, the intratracheal administration of phages did not induce upregulation of these markers in lung tissue, thereby confirming its safety profile and preventing the immune activation that leads to exhaustion [46].

Together with the evidence discussed above, this strongly supports the view that bacteriophages can be administered safely as antimicrobial agents, without imposing an additional inflammatory burden on the host in the long run.

### 3.2. Heterogeneity of Immune Response

A primary finding of this study is the pronounced lack of a uniform immune response pattern to phage therapy across the patient’s cohort. Unlike the uniform activation triggered by pathogenic viruses, exposure to therapeutic phage preparations did not induce significant, cohort-wide changes in the expression of key immune mediators. Instead, we observed highly individualized transcriptional profiles that appear to be shaped by the specific clinical context and outcome. Given this heterogeneity, we propose that these immunological profiles do not represent a direct, universal pro- or anti-inflammatory effect of the bacteriophages themselves. Rather, they likely reflect each patient’s unique underlying immune status, shaped by their ongoing chronic bacterial infection and other clinical factors, more than a direct effect of the therapy.

Among the host-specific factors examined, biological age emerged as a critical determinant of the response. Specifically, in the PBMC compartment, older patients exhibited a “primed” innate immune profile. Stratified analysis confirmed a significant upregulation of *TLR4* in this group compared to younger individuals. Furthermore, *MYD88*, the central adaptor protein for TLR signaling, showed a trend towards upregulation. This coordinated elevation of the *TLR4*-*MYD88* axis is consistent with the concept of “inflammaging”—a chronic, low-grade systemic inflammation characteristic of aging [23,24]. Recent evidence suggests that aging-associated oxidative stress and accumulated damage-associated molecular patterns (DAMPs) lower the activation threshold of these sensors [47,48], potentially explaining the heightened transcriptional responsiveness of innate machinery we observed in the older cohort. However, this age-associated ‘priming’ observed in mononuclear cells appears to be compartment-specific. In contrast to the PBMCs, granulocytes in older patients did not show a corresponding upregulation of sensing or effector modules. This dichotomy suggests that the burden of age-related immune dysregulation in these patients may be disproportionately carried by the mononuclear system, while granulocyte transcriptional plasticity remains relatively distinct.

The observed heterogeneity should not be interpreted as a confounding factor, but rather as a central characteristic suggesting that the immune response to phages is modulated by patient-specific immunological contexts rather than a universal mechanism. This aligns with methodological insights from other complex chronic diseases. Recent analyses of the European CRS Outcome Registry (CHRINOSOR) by Cavaliere et al. (2025) highlight substantial phenotypic and endotypic heterogeneity, demonstrating that clinical and molecular markers frequently dissociate even within large cohorts [49]. Our real-world data support a similar phenomenon in phage therapy: the immune response appears individualized and largely dependent on the patient’s pre-existing inflammatory status.

This interaction is further complicated by the patient’s individual microbiota and pre-existing “phageome.” It is possible that circulating phage particles are rapidly opsonized by neutralizing antibodies (induced or pre-existing), thereby masking them from innate recognition receptors on PBMCs [50]. Crucially, recent large-scale studies demonstrate that the presence of such antibodies does not necessarily preclude clinical efficacy [18,51], suggesting that therapeutic success is driven by local bacterial lysis rather than systemic immune activation. Future studies should consider profiling the patient’s baseline phageome to better elucidate these individualized interactions. Apart from direct phage–host interactions, the observed lack of response may also stem from immune exhaustion and metabolic changes in immune cells, particularly in the context of chronic bacterial infections [52].

Hierarchical clustering revealed a fundamental functional dichotomy between mononuclear and polymorphonuclear compartments. In PBMCs, we identified a transcriptional ‘uncoupling’ where innate sensor genes (*TLR2*, *TLR4*, *TLR10*, *NOD1*) were segregated in a separate cluster from effector cytokine genes. This topology suggests that detection of phage-associated molecular patterns does not obligately trigger a systemic inflammatory cascade. This observation aligns with functional data from Pincus et al., who demonstrated that while phage application could induce local inflammation in skin lesions, it failed to elicit pro-inflammatory cytokine production in isolated human PBMCs [53]. Such compartmentalization is further consistent with the concept of ‘inflammation anergy’, where tissue-resident macrophages limit pro-inflammatory output to prevent immunopathology [54]. These cells function as a physiological ‘firewall’—retaining phagocytic activity while limiting the release of pro-inflammatory cytokines to prevent systemic immunopathology [55]. Crucially, this is consistent with the danger model of immunity: since therapeutic phages do not cause cytotoxic damage to host tissues, enteral or topical administration of bacterial lysates alone does not generate sufficient danger signals to cross this activation threshold required for a systemic response [56]. Furthermore, the chronic nature of infections in our cohort may drive an innate immune reprogramming in circulating leukocytes that parallels the phenomenon of ‘endotoxin tolerance’—classically observed after repeated exposure to endotoxin—in which sustained stimulation with bacterial antigens induces a refractory, hyporesponsive state [46,57].

Our results highlight a critical distinction: although the adaptive immune system can recognize therapeutic phages—for example, by generating phage-neutralizing antibodies—the innate immune compartment is not hyperstimulated to mount a deleterious cytokine storm–like response, such as massive upregulation of IL-6 or TNF signaling. In addition, they underscore the distinct biological roles of these compartments in terms of efficacy: whereas PBMCs exhibited a “regulatory brake”, granulocytes displayed a “hard-wired” network (Cluster D) that couples pathogen sensing directly to rapid inflammatory signaling. This supports a model—recently corroborated by Weissfuss et al.—in which therapeutic phages primarily engage local “first responders” (neutrophils) without breaching the systemic activation threshold [58].

### 3.3. Study Limitations

Several limitations of this study must be acknowledged. The absence of a healthy control group receiving phage therapy restricts our ability to distinguish phage-specific immune effects from infection-related background activity. On the other hand, for this mechanistic study a self-controlled design has some advantages. It reduces the impact of inter-individual heterogeneity, increases statistical power by focusing on within-patient changes [59,60].

The cohort itself was heterogeneous with respect to age, infecting pathogens, clinical diagnosis, and treatment duration—an inherent feature of real-world personalized phage therapy. Although stratified analyses revealed age- or pathway-specific transcriptional trends, the exploratory sample size limits the robustness of subgroup comparisons. This limitation is consistent with findings from larger retrospective studies, where high biological and clinical variability constrained inferential statistics even in cohorts exceeding one hundred patients [51].

The observed molecular complexity arises within a highly diverse clinical context. Our cohort is heterogeneous with respect to age, pathogens, phages used, route of administration, and treatment duration—features that are intrinsic to real-world personalized phage therapy [51]. Thus, the correlations we describe should be viewed as hypothesis-generating and in need of confirmation in larger, stratified cohorts. At the same time, our molecular findings offer a mechanistic basis for the favorable safety profiles reported in larger series [51,61]. Furthermore, the profound heterogeneity of salvage-therapy patients challenges the rigid conventions of classical randomized controlled trials, and, as emphasized by Pirnay and Verbeken, a “one-size-fits-all” trial design is often incompatible with the inherently personalized nature of bacteriophage therapy [62].

Conducting this work in a salvage-therapy setting also introduced other practical limitations, including variation in sampling time. These deviations were unavoidable, as clinical care necessarily took precedence over the rigid procedural uniformity typical of RCTs. While they offer statistical power, they rarely incorporate the type of high-resolution immunological profiling used here. Implementing such deep molecular monitoring in a real-world environment is logistically demanding, yet it provides mechanistic insights often inaccessible in standard clinical workflows.

Biological constraints further shaped the study. Restricting analyses to peripheral blood prevented a direct assessment of tissue-resident immune responses, particularly those involving macrophages or microbiome-associated immunity in the gut. Future studies incorporating microbiome analysis and single-cell approaches will be essential to clarify compartment-specific processes and differentiate the behavior of discrete myeloid subsets. Another determinant of the immune response is the biological nature of the administered phages. Murine studies demonstrate that immunogenicity can differ markedly even between closely related T3 and T7 phages, underscoring the influence of phage identity, structural variation, and preparation method [63]. Because our patients received individualized cocktails comprising heterogeneous phages—without stratification by taxonomic family or batch-specific properties—we cannot fully distinguish host-related variation from phage-specific immunogenic differences.

Ideally, comparative analysis with intravenous administration would be beneficial; however, systemic delivery—while offering high bioavailability—is limited by rapid clearance and the induction of neutralizing antibodies [64]. Consequently, intravenous administration was not feasible in our clinical setting due to the safety and logistical profiles of patient-administered phage lysates.

Finally, the therapeutic agents in this study were sterile bacteriophage lysates prepared in nutrient broth and containing residual bacterial degradation products. While these data should be interpreted with some caution, they are internally consistent and biologically plausible. The favorable clinical tolerability offers real-world evidence on systemic activation thresholds, and the molecular findings corroborate this safety profile: despite the theoretical risk associated with bacterial remnants, no systemic inflammatory activation was observed.

### 3.4. Implications for Future Research

Our findings should be interpreted within the context of the urgent need for robust clinical data to support the widespread implementation of phage therapy. The history of biological therapies—most notably the cytokine-release reactions to Theralizumab—underscores the necessity of detailed immune characterization for novel agents [65]. Against this background, our study provides the first systematic, gene expression–based characterizations of the systemic immune response to personalized phage therapy in humans with chronic bacterial infections. In this regard, our study offers valuable molecular proof of concept. Specifically, it adds a mechanistic layer to prior observational safety data: it supports the favorable safety profile reported in larger observational studies and suggests that potential of phages to induce pro-inflammatory cytokines in vitro does not necessarily translate into systemic toxicity in a clinical setting [17,22,62,66].

Based on our exploratory data, we propose a refined, two-tiered strategy for molecular monitoring in future trials designed to elucidate phage–immune system interactions. First, we suggest that high-resolution monitoring of innate sensing modules (e.g., TLR4, MYD88) in PBMCs could serve to confirm the stability of the immune system during treatment. Crucially, such monitoring should be stratified by age. Second, with respect to efficacy assessment, future endpoints should explore the concept of “immune resolution.” Our observations highlight potential candidate markers, such as the downregulation of neutrophil-recruiting chemokines (e.g., CXCL8) and tissue-tolerance factors (e.g., GDF15), which may reflect the restoration of homeostasis.

Moreover, the discrepancy between the local bacterial lysis expected in phage therapy and the absence of systemic immune activation suggests that immunomodulatory events may be highly localized [53]. Relying solely on peripheral blood markers may therefore miss these local dynamics. Consequently, we recommend that future investigations implement concurrent sampling of the infection site alongside peripheral blood to capture the full scope of the host–phage–pathogen interaction.

Finally, taking into consideration accumulating evidence that combination therapy with antibiotics and phages is likely more effective than stand-alone phage therapy, future research should also address the impact of antibiotics on phage–immune system interactions [66,67].

## 4. Materials and Methods

### 4.1. Patients

Patients included in this study were selected from individuals suffering from chronic refractory bacterial infections qualified for the personalized experimental phage therapy program conducted at the Phage Therapy Unit in Wrocław. The program was conducted as a therapeutic experiment in accordance with a protocol formally approved by the competent bioethics committee and in full compliance with Polish legal regulations and the principles of the Declaration of Helsinki [22,68].

Patients were treated with sterile phage lysates containing individually selected bacteriophages (10^6^–10^9^ PFU/mL) from the therapeutic phage collection of the Bacteriophage Laboratory at the Hirszfeld Institute active against the target pathogen(s) in peptone water, LB broth or enrichment broth (also containing their bacterial metabolic and disintegration products). The phage formulations were prepared by the Bacteriophage Laboratory and administered orally, topically, and/or via inhalation [22,68]. Oral treatment involves 5–10 mL of phage preparation administered three times daily. Inhalation was performed twice daily with 2.5–5 mL of the formulation. Topical application was performed twice daily, including wound or skin lesion dressings, nasal drops/instillations (1 mL per nostril), and ear drops/rinses (1.5 mL). Intrarectal administration was applied twice daily at a dose of 10 mL. The choice of administration route for the phage preparations was determined according to the PT protocol individually for each patient by the supervising physician, based on the specific clinical presentation and the site of infection to optimize the potential therapeutic outcome [54]. No concomitant antibiotics or other antibacterial agents were used during the observation period of PT.

The clinical effects of PT were assessed for the respective day of therapy by a supervising physician using the A–G clinical PT score (Table 1), developed by Międzybrodzki et al. [22], which takes into account the results of follow-up microbiological cultures, changes in laboratory parameters (including white blood cell count—WBC, C-reactive protein—CRP, and erythrocyte sedimentation rate—ESR, usually evaluated up to 10 days before beginning of PT and up to 3 days before follow-up visit), and the severity of infection-related symptoms.

### 4.2. Isolation of Peripheral Blood Mononuclear Cells and Granulocytes

Venous blood samples were collected into heparin-tubes from patients on the day of PT initiation and again between days 13 and 29 of active treatment, in accordance with the therapeutic experiment protocol and the patients’ scheduled follow-up visits, as determined by their individual clinical course. Immediately after collection, the samples were processed for cell isolation. Granulocytes and PBMCs were isolated using a one-step double-density gradient centrifugation method [69]. Specifically, 4 mL of blood was gently layered onto 3 mL of Histopaque-1077 over 3 mL of Histopaque-1119 (both Sigma-Aldrich, St. Louis, MO, USA) in 15 mL conical tubes. Samples were centrifuged at 700× *g* for 25 min at room temperature. Cells from the upper interface between plasma and Histopaque-1077 (PBMCs) and from the lower interface between Histopaque-1077 and Histopaque-1119 (granulocytes) were collected separately and washed three times in phosphate-buffered saline (500× *g*, 5 min, room temperature).

Cell viability and morphology were assessed using simultaneous acridine orange (15 µg/mL) and ethidium bromide (50 µg/mL) staining under a fluorescence microscope [70,71,72]. Cell viability exceeded 95%, while PBMCs and granulocyte purities were greater than 98% and 96%, respectively. Remaining cell pellets were preserved in RNAlater (Invitrogen, Thermo Fisher Scientific, Waltham, MA, USA) and stored at −20 °C until RNA extraction.

### 4.3. RNA Extraction and Gene Expression Quantification

Total RNA was extracted using the RNeasy Mini Kit (Qiagen, Hilden, Germany) following the manufacturer’s protocol. RNA concentration and purity were assessed using a NanoDrop spectrophotometer (NanoDrop 2000, Thermo Fisher Scientific).

Expression levels of genes of interest were quantified by RT-qPCR using a custom RT^2^ Profiler PCR Array (Qiagen) on a CFX96 Real-Time PCR Detection System (Bio-Rad, Hercules, CA, USA), according to the manufacturer’s instructions. The analyzed panel comprised the following genes: *NFKB1* (nuclear factor kappa B subunit 1), *TLR2* (Toll-like receptor 2), *TLR4* (Toll-like receptor 4), *TLR10* (Toll-like receptor 10), *NOD1* (nucleotide-binding oligomerization domain-containing protein 1), *MYD88* (myeloid differentiation primary response 88), *MPO* (myeloperoxidase), *CXCL8* (C-X-C motif chemokine ligand 8), *CSF2* (colony stimulating factor 2), *GDF15* (growth differentiation factor 15), *DEFB1* (defensin beta 1), *IFNG* (interferon gamma), *IL10* (interleukin 10), *IL17A* (interleukin 17A), *IL1B* (interleukin 1 beta), *IL6* (interleukin 6), *IL4* (interleukin 4), *IL2* (interleukin 2), *IL5* (interleukin 5), *IL13* (interleukin 13), *IL21* (interleukin 21), and *TNF* (tumor necrosis factor). Two housekeeping genes, *ACTB* (actin beta) and *GAPDH* (glyceraldehyde-3-phosphate dehydrogenase), were included for normalization. Control procedures recommended by the array manufacturer were used to verify the absence of genomic DNA contamination, assess PCR inhibitor absence, and confirm the efficacy of RNA transcription.

In parallel, a third housekeeping gene, *G6PD* (glucose-6-phosphate dehydrogenase), was quantified as an additional endogenous control in a separate probe-based assay run on the same cDNA. This reaction was performed using the SensiFAST Probe No-ROX Kit (Meridian Bioscience, Cincinnati, OH, USA) according to the manufacturer’s protocol.

Relative transcript levels were calculated using the ΔΔCt method following the RT^2^ Profiler PCR Array guide (Qiagen). All reactions were performed in duplicates, and the mean cycle threshold value (Ct) was used. Gene expression levels were normalized to the average Ct of the three housekeeping genes. Fold change (FC) in gene expression between pre- and during-treatment samples was calculated for each patient using the 2^−ΔΔCt^ formula. A FC threshold ≤ 0.5 or ≥2 was used for identifying significantly altered gene expression [73].

### 4.4. Statistical Analysis

Statistical analysis was conducted using Statistica v.13 (TIBCO Software Inc., Palo Alto, CA, USA). The Wilcoxon signed-rank test (non-parametric) was applied to compare ΔΔCt values between time points, as data did not follow a normal distribution in the Shapiro–Wilk test [74,75]. Differences in FC values between groups defined by clinical parameters (sex, pathogen type, route of administration, age category, and short-versus long-term therapy) were assessed using the Mann–Whitney U test or Kruskal–Wallis ANOVA followed by Dunn’s post hoc test, as appropriate. Correlations between FC values and clinical variables (e.g., age, duration of PT, therapeutic response, inflammatory marker levels) were evaluated using Spearman’s rank correlation coefficient. For all analyses, a *p*-value < 0.05 was considered statistically significant.

Hierarchical gene clustering was performed separately for PBMCs and granulocytes using Ward’s minimum variance method with Euclidean distance, based on log_2_FC values for the analyzed genes. Clustering was performed without additional standardization so that the biological magnitude of the transcriptional response remained the primary weighting factor. This approach prioritized grouping genes according to the absolute magnitude of regulation rather than solely the similarity of their expression profiles. Distinct gene clusters identified in the dendrograms were designated as functional transcriptional modules.

### 4.5. Ethics Statement

The study was conducted in accordance with the Declaration of Helsinki and approved by the Bioethics Committee at Wrocław Medical University (protocol title: Immunomodulatory Effects of Bacteriophages on Immune Cells, approval no. KB–529/2021, issued on 17 June 2021).

## 5. Conclusions

To our knowledge, this is the first study to comprehensively analyze immune gene expression profiles in patients undergoing personalized PT. Within the limitations of this exploratory study, our results suggest that phage administration, unlike many viral infections, does not elicit a uniform or predictable systemic inflammatory response across the general population. This observation supports the overall safety profile of the enteral and/or topical PT, confirming the absence of a generalized overexpression of “cytokine storm” genes in peripheral blood leukocytes. However, we identified biological age as a potential determinant of the host response. Therefore, future clinical studies assessing immune responses should prioritize age as a significant variable differentiating host reactivity. Consequently, these findings indicate that the immunological outcome of PT is highly individualized by host- and phage-related biological factors, with any generalized phage-related immune effects likely modulated by the patient’s immune status and the consequences of phage-induced bacterial lysis. Elucidating these interactions between phages and the immune system is essential to uncover predictive markers that allow for better treatment stratification and the minimization of potential side effects.

We believe that this work helps to bridge the gap between phage therapy carried out as experimental treatment and formal clinical trials (none of which have so far proved successful, in contrast to the positive outcomes reported from compassionate-use cases) and provides essential insights and hypotheses to guide the rational design of future preclinical and clinical investigations.

## Figures and Tables

**Table 1:** Clinical data of analyzed patient cases, with the clinical effect of PT assessed according to Międzybrodzki et al. [22].

Patient No.	Gender	Age (years)	Pathogens (Targets for PT)	Diagnosis	Route of Administration of the Phage Preparation	Before PT			During PT			Day of PT	Clinical Effect of PT
						WBC (×1000/µL)	CRP (mg/L)	ESR (mm/h)	WBC (×1000/µL)	CRP (mg/L)	ESR (mm/h)		
1	F	33	*E. coli*	Chronic urinary tract infection. Neurogenic bladder.	Oral	7.8	7.14	12	9.4	29.7	44	21	F
2	M	38	*E. hormaechei*	Right hip infection	Oral	3.9	7.7	21	5	6.7	16	25	F
3	M	71	*K. pneumoniae*	Chronic bacterial prostatitis	Intrarectal	5.5	1.4	3	nd	nd	nd	22	C
4	M	27	*S. aureus*	Atopic dermatitis complicated by bacterial infection	Oral Topical	10.1	2.8	35	10	1	25	21	C
5	M	21	*P. aeruginosa*	Bronchitis. Kartagener’s syndrome.	Oral Inhalation	7.68	1	13	11.91	14.27	9	13	F
6	F	57	*S. aureus*	Chronic rhinosinusitis	Nasal drops Inhalation	8.17	nd	18	7.85	6.9	12	21	C
7	F	26	*P. aeruginosa* *S. aureus* *E. coli*	Otitis externa (bilateral)	Topical	6.8	3	15	6.25	2.9	nd	14	E
8	M	79	*S. aureus*	Surgical site infection after hernia mesh reapir. Diabetes t. II.	Oral Topical	6.94	9.5	16	7.35	9.3	13	29	D
9	F	67	*P. aeruginosa* *S. aureus*	Chronic leg ulcer	Topical	3.83	2.35	51	5.47	5.39	53	15	G
10	F	64	*P. aeruginosa*	Diabetic foot. Rheumatoid arthritis.	Oral Topical	7.61	84.1	60	9.01	61.5	32	14	C

Legend: PT—phage therapy; F—female; M—male; WBC—white blood cell count; CRP—C-reactive protein serum concentration; ESR—Erythrocyte Sedimentation Ratio; C—clinical improvement; nd—not done; D—questionable clinical improvement; E—transient clinical improvement; F—no response to treatment; G—clinical deterioration.

**Table 2:** Summary of fold-change values (mean, median, maximum, and minimum) in gene expression analyzed in PBMCs and granulocytes isolated from 10 patients undergoing experimental phage therapy. Mean or median values less than 0.5 or greater than 2.0 are highlighted in bold.

Pos.	Gene	PBMC				Granulocytes			
		Mean	Median	Min	Max	Mean	Median	Min	Max
1	*TNF*	1.48	1.15	0.36	5.35	1.14	0.87	0.36	3.27
2	*IL1B*	**3.50**	1.28	0.14	15.30	1.20	0.95	0.45	3.21
3	*IL17A*	**6.49**	1.97	0.05	48.67	1.95	0.64	0.12	10.33
4	*IL2*	**4.17**	**2.77**	0.03	23.26	1.07	0.86	0.17	2.90
5	*IL6*	**5.23**	1.51	0.14	32.11	1.56	0.92	0.12	4.24
6	*IL21*	**2.38**	1.06	0.30	13.78	1.58	0.89	0.41	5.75
7	*IFNG*	1.73	1.24	0.11	7.21	**2.07**	1.45	0.17	5.89
8	*CXCL8*	**2.18**	**2.04**	0.04	4.70	0.93	0.87	0.09	3.12
9	*CSF2*	1.92	1.25	0.15	6.97	1.74	1.23	0.23	4.31
10	*MPO*	**2.44**	1.14	0.30	10.87	**2.44**	0.76	0.19	12.76
11	*NFKB1*	1.09	1.18	0.72	1.42	0.98	0.88	0.78	1.94
12	*NOD1*	1.32	0.95	0.40	3.62	1.53	0.83	0.38	6.23
13	*MYD88*	1.17	1.12	0.69	1.84	1.01	0.97	0.57	1.65
14	*TLR2*	1.11	0.93	0.70	1.91	0.92	0.89	0.25	2.21
15	*TLR4*	1.13	1.20	0.79	1.40	0.89	0.96	0.41	1.34
16	*TLR10*	1.20	1.21	0.32	2.24	1.07	0.85	0.50	2.93
17	*GDF15*	**2.59**	1.26	0.20	11.09	**2.37**	1.25	0.21	9.05
18	*DEFB1*	**3.84**	1.26	0.14	18.85	**3.78**	0.78	0.24	13.91
19	*IL5*	**4.67**	1.95	0.08	31.67	1.74	1.35	0.17	4.40
20	*IL13*	**5.13**	1.78	0.02	27.19	1.60	0.87	0.12	6.84
21	*IL4*	**4.25**	1.63	0.04	28.74	**2.18**	0.88	0.16	8.13
22	*IL10*	**4.67**	1.65	0.15	31.12	1.97	1.11	0.25	5.50

## Data Availability

The original contributions presented in this study are included in the article. Further inquiries can be directed to the corresponding authors.

## Abbreviations

The following abbreviations are used in this manuscript:

CRP	C-reactive protein
CSF2	Colony stimulating factor 2
CXCL8	C-X-C motif chemokine ligand 8
DEFB1	Defensin beta 1
ESR	Erythrocyte sedimentation rate
FC	Fold change
GDF15	Growth differentiation factor 15
HCV	Hepatitis C virus
HHV-6	Human herpesvirus 6
HSV-1	Herpes simplex virus 1
IFNG	Interferon gamma
IL10	Interleukin 10
IL13	Interleukin 13
IL17A	Interleukin 17A
IL1B	Interleukin 1 beta
IL2	Interleukin 2
IL21	Interleukin 21
IL4	Interleukin 4
IL5	Interleukin 5
IL6	Interleukin 6
MAPK	Mitogen-activated protein kinase
MPO	Myeloperoxidase
MYD88	Myeloid differentiation primary response 88
NF-κB	Nuclear factor kappa-light-chain-enhancer of activated B cells
NFKB1	Nuclear factor kappa B subunit 1 gene
NOD1	Nucleotide-binding oligomerization domain-containing protein 1
PFU	Plaque-forming unit
PBMC	Peripheral blood mononuclear cell
PRRs	Pattern-recognition receptors
PT	Phage therapy
RCT	Randomized controlled trial
RSV	Respiratory syncytial virus
TLR	Toll-like receptor
TNF	Tumor necrosis factor
WBC	White blood cell
